# The cell competition-based high-throughput screening identifies small compounds that promote the elimination of RasV12-transformed cells from epithelia

**DOI:** 10.1038/srep15336

**Published:** 2015-10-20

**Authors:** Hajime Yamauchi, Takanori Matsumaru, Tomoko Morita, Susumu Ishikawa, Katsumi Maenaka, Ichigaku Takigawa, Kentaro Semba, Shunsuke Kon, Yasuyuki Fujita

**Affiliations:** 1 Division of Molecular Oncology, Institute for Genetic Medicine, Hokkaido University Graduate School of Chemical Sciences and Engineering, Sapporo, Japan; 2 Center for Research and Education on Drug Discovery, Faculty of Pharmaceutical Sciences, Hokkaido University, Sapporo, Japan; 3 Laboratory of Biomolecular Science, Faculty of Pharmaceutical Sciences, Hokkaido University, Sapporo, Japan; 4 Hokkaido University Graduate School of Information Science and Technology, Sapporo, Japan; 5 Department of Life Science and Medical Bioscience, School of Advanced Science and Engineering, Waseda University, Tokyo, Japan; 6 Division of Gene Function Analysis, Translational Research Center, Fukushima Medical University, Fukushima, Japan

## Abstract

Recent studies have revealed that cell competition can occur between normal and transformed epithelial cells; normal epithelial cells recognize the presence of the neighboring transformed cells and actively eliminate them from epithelial tissues. Here, we have established a brand-new high-throughput screening platform that targets cell competition. By using this platform, we have identified Rebeccamycin as a hit compound that specifically promotes elimination of RasV12-transformed cells from the epithelium, though after longer treatment it shows substantial cytotoxic effect against normal epithelial cells. Among several Rebeccamycin-derivative compounds, we have found that VC1-8 has least cytotoxicity against normal cells but shows the comparable effect on the elimination of transformed cells. This cell competition-promoting activity of VC1-8 is observed both *in vitro* and *ex vivo*. These data demonstrate that the cell competition-based screening is a promising tool for the establishment of a novel type of cancer preventive medicine.

At the initial stage of carcinogenesis, transformation occurs in single cells within epithelial layers. However, it remains elusive what happens at the interface between normal cells and the newly emerging transformed cells during this process. Recent studies have revealed that normal and transformed epithelial cells often compete with each other for cell survival: a phenomenon called cell competition. Cell competition between normal and transformed epithelial cells was originally reported in *Drosophila*^1^,^2^,^3^,^4^,^5^, and has been recently shown to occur in mammals as well^6^,^7^. For example, when oncoprotein Ras-, Src-, or ErbB2-transformed cells are surrounded by normal epithelial cells, the transformed cells are occasionally extruded from the apical surface of the normal epithelial monolayer^8^,^9^,^10^,^11^. In addition, when tumor suppressor protein Scribble- or Mahjong-transformed cells are surrounded by normal epithelial cells, the transformed cells undergo apoptosis and eventually leave the epithelium^12^,^13^. Furthermore, a recent report has demonstrated that normal epithelial cells have an ability to recognize and actively eliminate the neighboring transformed cells, indicating that normal epithelial tissues are endowed with anti-tumor activity that does not involve immune systems^14^. This process is named EDAC (Epithelial Defense Against Cancer).

Since the discoveries of oncogenes and tumor suppressor genes, oncologists have elucidated how mutations of these genes affect signaling pathways and behavior of cells and clarified the differences between normal and transformed cells. Accordingly, by intensive high-throughput compound screenings that target key molecules involved in those signaling pathways, a number of chemical compounds have been identified that specifically eradicate transformed cells. Indeed, by this strategy, several drugs have been successfully invented, and some of them are now used in a clinical scene. However, in most cases it is difficult to specifically affect only transformed cells, and the side effect on normal cells often prevents these drugs from clinical usage. Therefore, a breakthrough has been awaited: a novel type of cancer treatment that involves a different mechanism of action, and thus does not cause such deleterious side effects.

In this study, we have established a unique high-throughput screening platform that targets cell competition between normal and transformed cells, an unexplored mechanism of action in the conventional cancer research. Using this platform, we have identified small chemical compounds that selectively promote the elimination of transformed cells from the epithelium.

## Results

### Identification of Rebeccamycin as a compound that enhances cell competition between normal and RasV12-transformed epithelial cells

**Figure 1:**
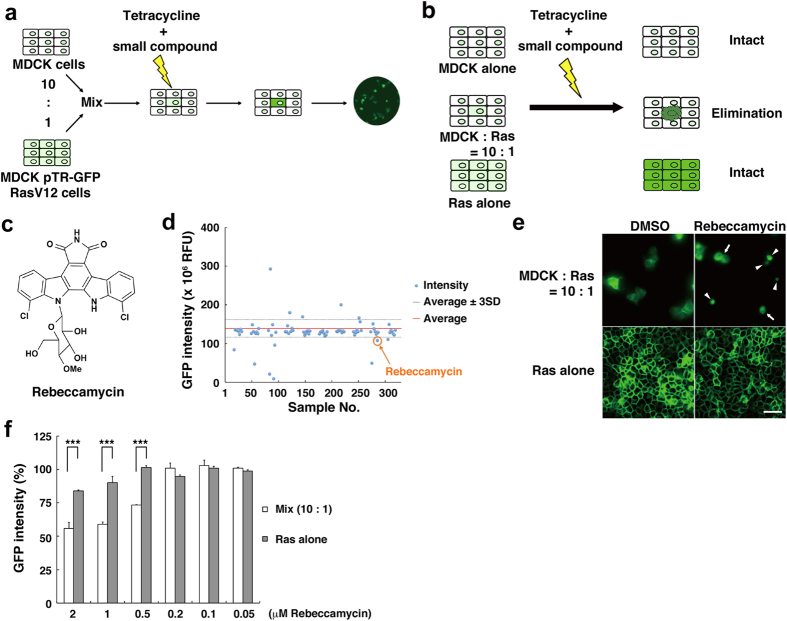
A novel type of high-throughput screening has lead to the identification of Rebeccamycin as a chemical compound that enhances cell competition between normal and transformed epithelial cells. (**a**) A scheme for the high-throughput screening platform we have developed. Normal MDCK cells were mixed with MDCK-pTR GFP-RasV12 cells at a ratio of 10:1 and seeded into a 96-well plate. The mixed cells were incubated for 16 h until a monolayer was formed. Then, the culture medium was exchanged for a new medium containing tetracycline and each small chemical compound, followed by incubation for 16 h. Finally, the GFP intensity of the remaining GFP-RasV12-transformed cells was quantified. (**b**) The secondary screening for small chemical compounds that specifically induces the elimination of RasV12-transformed cells that are surrounded by normal cells. (**c**) A structural formula of Rebeccamycin. (**d**) An example of the primary screening result including Rebeccamycin (orange circle). The blue dot shows the fluorescence intensity of MDCK-pTR GFP-RasV12 cells after treatment with each compound. The red line and black dot lines indicate the average value and the 3-fold standard deviation (SD) above or below the average, respectively. (**e**) Fluorescence images of MDCK-pTR GFP-RasV12 cells that are surrounded by MDCK cells (upper panels) or cultured alone (lower panels) with DMSO (left) or 2 μM Rebeccamycin (right). Arrows and arrowheads indicate cells that have become round and small, and been broken into pieces, respectively. Scale bar: 50 μm. (**f**) Quantification of the GFP intensity of MDCK-pTR GFP-RasV12 cells mixed with MDCK cells (white bar) or cultured alone (gray bar) with the indicated concentration of Rebeccamycin. Values are expressed as a ratio relative to DMSO treatment. Data are mean ± SD from three independent experiments. ***P < 0.001.

Madin-Darby canine kidney (MDCK) cells are non-transformed epithelial cells that have been widely used for studying epithelial biology, because they retain characteristics of functional epithelia^15^. We have previously established MDCK cells stably expressing a constitutively active mutant of Ras (RasV12) in a tetracycline-inducible manner (MDCK-pTR GFP-RasV12 cells)^10^. Ras is a prototype-oncoprotein that is frequently mutated to its active forms in various types of human cancers^16^. Using this cell line, we have demonstrated that when RasV12-transformed cells are surrounded by normal epithelial cells, the transformed cells are occasionally extruded from the apical surface of an epithelial monolayer^10^ and that the surrounding normal epithelial cells are actively involved in the elimination of transformed cells^14^. By applying this cell competition between normal and RasV12-transformed cells, we have developed the following screening platform (Fig. 1a). First, normal MDCK cells and MDCK-pTR GFP-RasV12 cells are mixed at a ratio of 10:1 and cultured in a 96-well plate until they form an epithelial monolayer. Then, a small chemical compound is added in each well, together with tetracycline to induce expression of GFP-RasV12. After several hours, the GFP fluorescence intensity is measured to quantify the remaining GFP-RasV12-expressing cells. We have optimized the cell numbers and incubation times, thereby creating a condition where transformed cells are surrounded by normal cells at high cell density. In the primary screening, we selected small compounds that enhance the exclusion of GFP-RasV12-positive cells from epithelia. In the following secondary screening, we compared the effect on RasV12-transformed cells surrounded by normal cells with that on RasV12 cells cultured alone, and selected small compounds that specifically eliminate the former cells (Fig. 1b). After screening with 2,607 small chemical compounds, we identified Rebeccamycin as a hit compound (Fig. 1c,d and Supplementary Table 1). Rebeccamycin is an antibiotic isolated from the actinomycete strain, *Saccharothrix aerocolonigenes*, which inhibits topoisomerase I and induces anti-tumor activity against various cancer cell lines^17^,^18^. For RasV12-transformed cells that were surrounded by normal cells, addition of Rebeccamycin induced morphological changes into round and small ‘stressed’ shapes (Fig. 1e; arrows), often leading to fragmentation (Fig. 1e; arrowheads). In contrast, this effect was not observed for RasV12 cells that were cultured alone (Fig. 1e) or normal cells (data not shown). This specific effect against RasV12 cells surrounded by normal cells was observed in a dose-dependent manner with a minimum effective dose at 0.5 μΜ (Fig. 1f). These data demonstrate that Rebeccamycin selectively affects RasV12-transformed cells that are surrounded by normal cells and enhances their elimination from the epithelium.

### VC1-8, an analogous compound of Rebeccamycin, has less cytotoxicity and comparable effect on the elimination of RasV12-transformed cells

**Figure 2:**
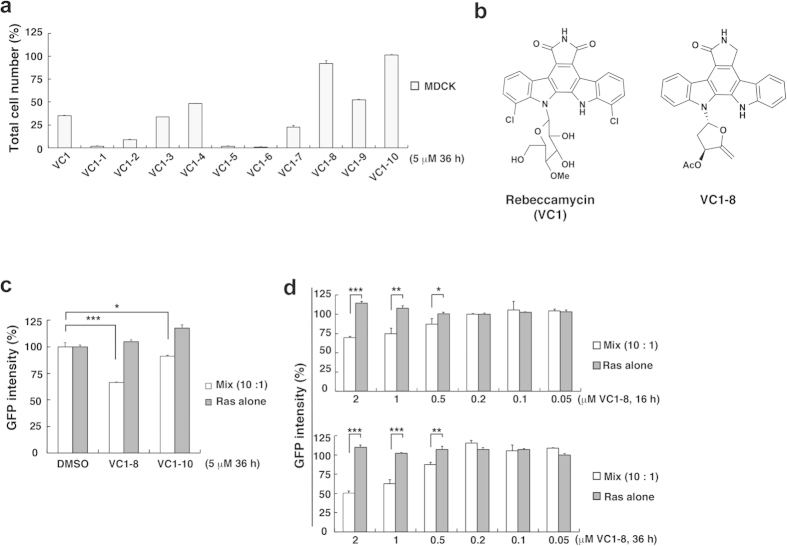
Rebeccamycin-derivative VC1-8 has significant cell competition-promoting activity with least toxicity against normal cells. (**a**) Cell survival ratio after treatment with Rebeccamycin (VC1) or its analogous compounds for 36 h. Data are mean ± SD from three independent experiments. Values are expressed as a ratio relative to DMSO treatment. (**b**) Structural formulae of Rebeccamycin (VC1) and VC1-8. The formulae of the other tested analogous compounds are shown in Supplementary Table 2a. (**c**) Effect of VC1-8 or VC1-10 on MDCK-pTR GFP-RasV12 cells mixed with MDCK cells (white bar) or cultured alone (gray bar). Data are mean ± SD from three independent experiments. Values are expressed as a ratio relative to DMSO treatment. *P < 0.05, ***P < 0.001. (**d**) Dose dependent effect of VC1-8 on MDCK-pTR GFP-RasV12 cells mixed with MDCK cells (white bar) or cultured alone (gray bar) after 16 h (top) or 36 h (bottom) treatment. Data are mean ± SD from three independent experiments. Values are expressed as a ratio relative to DMSO treatment. *P < 0.05, **P < 0.01, ***P < 0.001.

Rebeccamycin is known to have substantial cytotoxic and water-insoluble property. Indeed, we found that after the longer incubation with Rebeccamycin (in this screening, we call Rebeccamycin as VC1 (validated compound library 1)), the survival ratio of normal MDCK cells was substantially suppressed (Fig. 2a and Supplementary Fig. S1a). Thus, to explore less toxic agents we next examined the cytotoxic property of VC1-analogous compounds (Supplementary Table 2a). Among 10 tested analogues, two compounds (VC1-8 and VC1-10) showed least cytotoxicity (Fig. 2a,b and Supplementary Fig. S1). In addition, VC1-8 specifically induced elimination of RasV12 cells surrounded by normal cells to the comparable extent as VC1, whereas VC1-10 showed less prominent effect (Fig. 2c). This effect of VC1-8 was observed in a dose-dependent manner with a minimum effective dose at 0.5 μΜ (Fig. 2d).

### VC1-8 induces cell death of RasV12-transformed MDCK cells surrounded by normal MDCK cells

**Figure 3:**
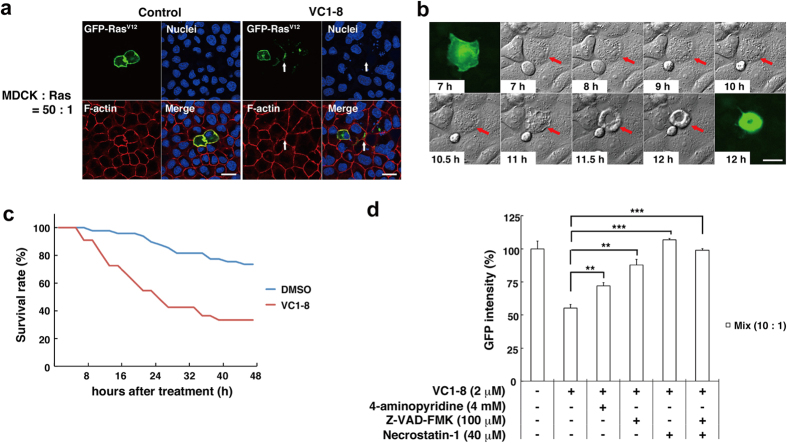
VC1-8 induces cell death of transformed cells that are surrounded by normal cells. (**a**) Immunofluorescence images of MDCK-pTR GFP-RasV12 cells that are surrounded by MDCK cells in the absence (left) or presence (right) of 2 μM VC1-8 for 16 h. Cells are stained with Hoechst 33342 (blue) and Alexa-Fluor-568-conjuated phalloidin (red). Arrows indicate a cell with fragmentation. Scale bars: 20 μm. (**b**) Images of a representative time-lapse analysis of MDCK-pTR GFP-RasV12 cells that are surrounded by MDCK cells (red) in the presence of 2 μM VC1-8. Scale bar: 20 μm. (**c**) A graph showing the survival rate of MDCK-pTR GFP-RasV12 cells surrounded by MDCK cells in the presence of DMSO (blue) or 2 μM VC1-8 (red). n = 49 cells (DMSO) and 33 cells (VC1-8). The effect of VC1-8 was statistically significant (*P* < 0.0001). (**d**) Effect of apoptosis inhibitor (4-aminopyridine, Z-VAD-FMK) and/or necroptosis inhibitor (Necrostatin-1) on MDCK-pTR GFP-RasV12 cells mixed with MDCK cells. Data are mean ± SD from three independent experiments. Values are expressed as a ratio relative to DMSO treatment. **P < 0.01, ***P < 0.001.

Next, we further analyzed the effect of VC1-8 on the cellular phenotype by microscopic analyses. Addition of VC1-8 induced cell death-like morphology; irregular cell shape with frequent fragmentation into small pieces (Fig. 3a). Time-lapse microscopic analyses revealed that the morphological changes initiated around 8–12 h after addition of VC1-8 (Fig. 3b), and the quantification of the time-lapse data showed that VC1-8 substantially enhanced the elimination of RasV12-transformed cells from the epithelial monolayer (Fig. 3c). Co-incubation with apoptosis inhibitor (4-aminopyridine or Z-VAD-FMK) and/or necroptosis inhibitor (Necrostatin-1) significantly diminished the effect of VC1-8, suggesting that VC1-8 mediates apoptosis and/or necroptosis of RasV12 cells surrounded by normal cells (Fig. 3d).

### VC1-8 also induces elimination of RasV12-transformed MCF10A epithelial cells

**Figure 4:**
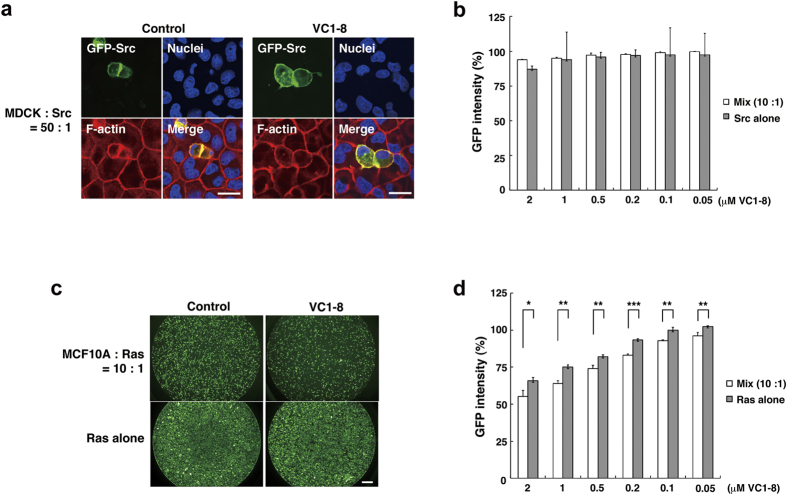
VC1-8 also enhances the elimination of human RasV12-transformed mammary epithelial cells. (**a**) Immunofluorescence images of MDCK-pTR GFP-cSrcY527F cells that are surrounded by MDCK cells in the absence (left) or presence (right) of 2 μM VC1-8. Cells are stained with Hoechst 33342 (blue) and Alexa-Fluor-568-conjuated phalloidin (red). Scale bars: 20 μm. (**b**) Dose-dependent effect of VC1-8 (16 h) on MDCK-pTR GFP-cSrcY527F cells mixed with MDCK cells (white bar) or cultured alone (gray bar). Data are mean ± SD from three independent experiments. Values are expressed as a ratio relative to DMSO treatment. (**c**) Fluorescence images of MCF10A-pTR GFP-RasV12 cells that are surrounded by MCF10A cells (top) or cultured alone (bottom) in the absence (left) or presence (right) of 2 μM VC1-8. Scale bar: 50 μm. (**d**) Dose-dependent effect of VC1-8 (24 h) on MCF10A-pTR GFP-RasV12 cells mixed with MCF10A cells (white bar) or cultured alone (gray bar). Data are mean ± SD from three independent experiments. Values are expressed as a ratio relative to DMSO treatment. *P < 0.05, **P < 0.01, ***P < 0.001.

Furthermore, we examined whether VC1-8 also induces the elimination of the other type of transformed cells. To this end, we used MDCK cells expressing GFP-cSrcY527F (oncogenic Src mutant) in a tetracycline-inducible manner (MDCK-pTR GFP-cSrcY527F cells)^19^. We found that addition of VC1-8 induced neither cell morphological change nor elimination of Src-transformed cells that were surrounded by normal cells (Fig. 4a,b), suggesting that VC1-8 specifically affects RasV12-transformed cells. Next, we examined the effect of VC1-8 on another RasV12-transformed cell line: MCF10A human mammary epithelial cells expressing GFP-RasV12 in a tetracycline-inducible manner (MCF10A-pTR GFP-RasV12 cells). We showed that treatment with VC1-8 decreased the number of RasV12-transformed cells surrounded by normal cells to the greater extent than that of RasV12 cells cultured alone, with a minimum effective dose at less than 0.05 μΜ (Fig. 4c,d), suggesting the general cell competition-enhancing effect of VC1-8 on RasV12-transformed epithelial cells.

### VC1-8 enhances the eradication of RasV12-transformed cells from the mouse intestinal epithelial monolayer

**Figure 5:**
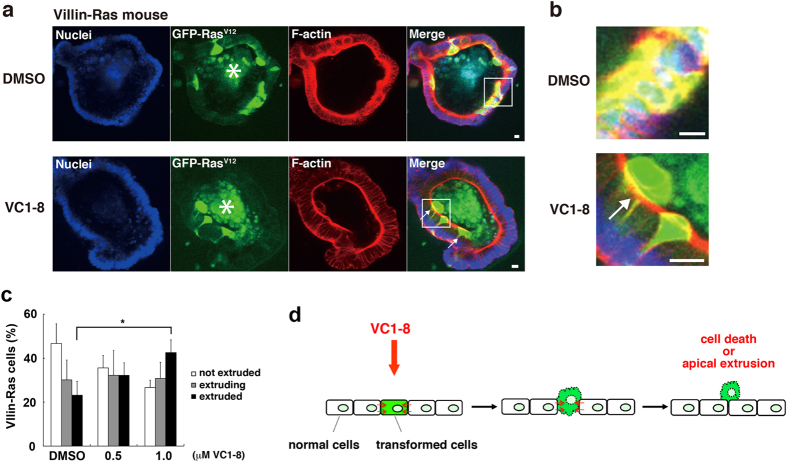
VC1-8 promotes the elimination of RasV12-transformed cells from the mouse intestinal epithelium. (**a**) Immunofluorescence images of RasV12- transformed cells in the intestinal epithelium of the mouse crypt *ex vivo* culture in the absence (top) or presence (bottom) of 1 μM VC1-8. Intestinal epithelial cells were collected from mice obtained by crossing loxP-stop-loxP-KrasV12-IRES-eGFP and Villin-Cre/ER^T2^ mice. After applying them into the organ crypt culture, tamoxifen was added to the grown crypts to induce a mosaic expression of RasV12 within the mouse intestinal epithelium. Cells are stained with Hoechst 33342 (blue) and Alexa-Fluor-568-conjuated phalloidin (red). Arrows indicate the apically extruded GFP-RasV12-expressing cells from the epithelium. The asterisks indicate mucin-rich autofluorescent materials in the apical lumen. Scale bar: 50 μm. (**b**) A magnified image of the apically extruded RasV12-transformed cells (from a white box in (**a**)). (**c**) Effect of VC1-8 on the frequency of apical extrusion of RasV12-transformed cells in the *ex vivo* system: not extruded (white bar), extruding (grey bar) or extruded (black bar) RasV12-transformed cells from an epithelial monolayer; extruding: cells moving apically but still attached to the basal membrane, extruded: cells apically extruded and detached from the basal membrane. Data are mean ± SD from three independent experiments. *P < 0.05. (**d**) A schematic model of the cell competition-promoting effect of VC1-8. VC1-8 enhances the interaction between normal and transformed epithelial cells: promoting the attacking force of normal cells against transformed cells or attenuating the defensive force of transformed cells, eventually leading to cell death or apical extrusion of transformed cells.

To further confirm the pharmacological effect of VC1-8, we used a newly established cell competition *ex vivo* culture system. First, a cell competition model mouse was obtained by crossing loxP-stop-loxP-KrasV12-IRES-eGFP and Villin-Cre/ER^T2^ mice, in which a low dose of tamoxifen induces the expression of RasV12 in a mosaic manner within the intestinal epithelium (Kon *et al.*, manuscript in preparation). The intestinal epithelial cells were collected from this mouse and were applied to the *ex vivo* organ crypt culture in the Matrigels^20^. Just prior to the formation of intestinal crypts, a low dose of tamoxifen was added into the culture medium to induce a mosaic expression of RasV12 within the epithelium. VC1-8 was then added, and the fate of RasV12-transformed cells that were surrounded by normal epithelial cells was analyzed after 36 h of VC1-8 treatment. As expected, even in the absence of VC1-8, the extrusion of RasV12 cells into the apical lumen of epithelia was observed at a certain ratio (Fig. 5a–c) (Kon *et al.*, manuscript in preparation). But, addition of VC1-8 further enhanced the apical elimination of the transformed cells significantly (Fig. 5a–c), suggesting that the cell competition-promoting effect of VC1-8 is also observed in the *ex vivo* system.

## Discussion

Recent studies have revealed that cell competition can occur between normal and transformed epithelial cells in mammals^6^,^7^. In this study, we have established the first screening platform that targets cell competition, and identified Rebeccamycin and VC1-8 as the chemical compounds that specifically promote the elimination of transformed cells from a monolayer of normal epithelial cells (Fig. 5d), demonstrating that cell competition can be applied into a novel type of cancer prevention and/or treatment. Rebeccamycin possesses anti-tumor activity against several cancer cell lines^17^,^18^, but has not been brought into clinical trials because of its water-insolubility and cytotoxicity. We have found that Rebeccamycin has an ability to promote cell competition between normal and RasV12-transformed epithelial cells, though it also shows substantial cytotoxicity against normal cells. Among several Rebeccamycin-derivatives, we have found that VC1-8 has least cytotoxicity against normal cells but shows the comparable cell competition-promoting effect. We also demonstrate that VC1-8 promotes elimination of transformed cells both *in vitro* and *ex vivo*. By further modifying the structure of VC1-8, we are now attempting to obtain even more potent compounds for the future clinical application. For another Rebeccamycin-derivative NSC65549 (also known as XL119 or Becatecarin), several Phase II clinical trials were conducted against various types of tumors including renal, colorectal and lung cancers^21^,^22^,^23^,^24^, but the overall outcome has not been clarified yet. The formula structure of VC1-8 is quite different from that of NSC65549 (Supplementary Table 2a,b), thus it is likely that those compounds target different molecules.

Recently, several key regulators of cell competition have been identified. Importantly, those regulators specifically function in either normal or transformed cells. For example, at the interface between normal and RasV12-transformed cells, activity and/or expression of EPLIN, Cav-1, and VASP are specifically modulated in RasV12-transformed cells^19^,^25^, whereas filamin and vimentin accumulate in normal cells at the boundary with the neighboring transformed cells and play a positive role in the elimination of transformed cells^14^. At present, it is not clear whether VC1-8 affects either normal or transformed cells, but to further understand the mode of action of VC1-8, the target cell(s) and molecule(s) need to be elucidated in future studies.

The high-throughput screening that we have developed in this study targets the interaction between transformed cells with a single oncogenic mutation and normal cells, which potentially illustrates the process occurring at the initial stage of carcinogenesis. Thus, the cell competition-based screening will potentially lead to a novel type of cancer preventive treatment; enhancing the attacking force of normal cells against transformed cells or attenuating the defensive force of transformed cells, thereby promoting eradication of newly emerging transformed cells from epithelia (Fig. 5d). There exist no schemes of clinical trials for such cancer preventive medicine at present, but by further advancing these studies on cell competition, we aim to establish a new research framework to develop unprecedented cancer preventive drugs. In addition, a recent study revealed that enhancement of cell competition could potentially prolong lifespan^26^, providing the cell competition-based screening with further broader applications.

## Methods

### Cell Culture

MDCK, MDCK-pTR GFP-RasV12, and MDCK-pTR GFP-cSrcY527F cells were cultured as previously described^10^,^19^. For tetracycline-inducible MDCK cell lines, 10 μg/ml tetracycline (Sigma-Aldrich) was used to induce the expression of GFP-RasV12 or GFP-cSrcY527F. MCF10A-Tet-on Ad cells^27^ were cultured in DMEM/F12 (Life Technologies) containing 5% Horse Serum (Life technology), 20 ng/ml EGF (BD bioscience), 0.5 μg/ml Hydrocortisone (Sigma-Aldrich), 100 ng/ml Cholera Toxin (List Biological Laboratories), 10 μg/ml Insulin (Sigma-Aldrich), 0.5 μg/ml G418 (Calbiochem), and 1% penicillin/streptomycin (Life Technologies). To establish MCF10A cells stably expressing GFP-RasV12 in a tetracycline-inducible manner, MCF10A-Tet-on Ad cells were transfected with pTRE-Tight-HC-EGFP-RasV12 and pBabe-puromycin using Lipofectamine^TM^ 2000 (Life Technologies), followed by selection in the medium containing 0.5 μg/ml G418 and 0.5 μg/ml puromycin (Sigma-Aldrich). For this cell line, 10 μg/ml doxycycline was added to induce the expression of GFP-RasV12.

For Figs 1, 2, 3b–d and 4b–d and Supplementary Fig. S1, cells were seeded on CellCarrier-96 Black, Optically Clear Bottom plates (PerkinElmer) at a density of 5.5 × 10^4^ cells and incubated for 16 h. Then, cells were incubated with tetracycline and a small chemical compound for the indicated times. For Figs 3a and 4a, cells were seeded on glass cover slips in a 6–well culture plate at a density of 7.0 × 10^5^ cells, followed by incubation with tetracycline for 16 h with or without 2 μM VC1-8. For Fig. 3d, inhibitors were added at the same time as VC1-8. The following inhibitors were used: 4-aminopyridine (4 mM, Sigma-Aldrich), Z-VAD-FMK (100 μM, Calbiochem) or Necrostatin-1 (40 μM, Sigma-Aldrich). DMSO (Sigma-Aldrich) was used as a control. For Fig. 5a–c, the mouse intestinal crypt culture was performed as previously described^20^. First, intestinal epithelial cells were collected from mice obtained by crossing loxP-stop-loxP-KrasV12-IRES-eGFP and Villin-Cre/ER mice, and were applied to the *ex vivo* organ crypt culture in the Matrigels. After 108 h, 100 nM tamoxifen (Sigma-Aldrich) was added to the grown crypts to induce a mosaic expression of RasV12 within the mouse intestinal epithelium (Kon *et al.*, manuscript in preparation). After 24 h, VC1-8 was added into the culture medium, and the phenotype was observed after 36 h of VC1-8 treatment. All animal experiments were conducted under the guidelines by the Animal Care Committee of Hokkaido University. The animal protocols were reviewed and approved by the Hokkaido University Animal Care Committee (Approval number:12-0116).

### Immunofluorescence

Alexa-Fluor-568-conjugated phalloidin (Life Technologies) was used at 1.0 U/ml. Hoechst 33342 (Life Technologies) was used at a dilution of 1:5,000. For immunofluorescence, MDCK-pTR GFP-RasV12 cells or MDCK-pTR GFP-cSrcY527F cells were mixed with MDCK cells at a ratio of 1:50, and cultured on glass cover slip as previously described^10^. The mixture of cells was incubated for 16 h, followed by tetracycline treatment for 16 h. Cells were fixed with 4% PFA in PBS, and immunofluorescence was performed as previously described^28^. Immunofluorescence images were analyzed by the Olympus FV1000 system and Olympus FV10-ASW software. Images were quantified by MetaMorph software (Universal imaging).

### Chemical Library

The small chemical compounds library was from Open Innovation Center for Drug Discovery, Tokyo University. This library consists of 2,607 validated compounds, including LOPAC^1280^ (Sigma-Aldrich) and Prestwick chemical compounds (Prestwick Chemical), which have known pharmacological activity (Supplementary Table 1).

### High-throughput screening

For the high-throughput primary screening, 5 × 10^4^ MDCK cells were mixed with 5 × 10^3^ MDCK-pTR GFP-RasV12 cells and seeded into a well on the CellCarrier-96 Black, Optically Clear Bottom plate. The mixed cells were incubated at 37 °C for 16 h until a monolayer was formed. Then, the culture medium was exchanged for a new medium containing 10 μg/ml tetracycline and 2 μM each small chemical compound using Drug Discovery Support Automatic Screening device Hornet-HTS (WAKO), followed by incubation for 16 h. Finally, cells were washed with PBS using Microplate Washer (Tecan), fixed in 4% paraformaldehyde/PBS, and stained with Hoechst 33342. The images of cells were captured by using Operetta High Content Imaging System (PerkinElmer), followed by the analysis of GFP intensity of MDCK-pTR GFP-RasV12 cells. For the primary screening, DMSO alone was added into 8 wells per plate as a positive control to set the average and standard deviation (SD). And, small compounds with GFP intensity outside the average ± 3 SD of the positive control were selected (please see Fig. 1d for a reference). In addition, the cytotoxic effect of compounds was also evaluated by counting the total cell number with the Hoechst staining. Chemical compounds that induced substantial decrease ( < 60%) in the total cell number was excluded from the secondary screening. For the secondary screening, the following three culture conditions were used for analyses; MDCK cells alone, MDCK-pTR GFP-RasV12 cells alone, and MDCK-pTR GFP-RasV12 cells mixed with MDCK cells at a ratio of 1:10. These cells were cultured and incubated with chemical compounds in the same way as the primary screening. For the secondary screening, we set the following cutoff points; 1) The GFP intensity ration (relative to DMSO control) of MDCK-pTR GFP-RasV12 cells mixed with MDCK cells was substantially lower than that of MDCK-pTR GFP-RasV12 cells cultured alone. 2) Cell viability of normal MDCK cells was > 60%. For the tertiary screening, the dose-dependent effect was examined for each compound. After screening 2,607 chemical compounds, we obtained only Rebeccamycin as a hit compound.

### Time-lapse microscopy

For time-lapse microscopy, 1.5 × 10^6^ MDCK cells were mixed with 3.0 × 10^4^ MDCK-pTR GFP-RasV12 cells and seeded on a 35-mm glass-bottom culture dish (Matsunami) as previously described^10^. The mixture of cells was incubated for 16 h, followed by addition of 10 μg/ml tetracycline and 2 μM VC1-8, and the time-lapse observation was started after 6 h.

### Data analyses

For data analyses, two-tailed Student’s *t*-tests were used to determine *P*-values, except for Fig. 3c where Long-rank test was used. For Figs 1, 2, 3 and 4, quantification of GFP intensity was performed using the Operetta High Content Imaging System. For Fig. 5a–c, apical extrusion of RasV12 cells was examined by using confocal microscopy. More than 50 RasV12 cells were analyzed for each experimental condition.

## Additional Information

**How to cite this article**: Yamauchi, H. *et al.* The cell competition-based high-throughput screening identifies small compounds that promote the elimination of RasV12-transformed cells from epithelia. *Sci. Rep.* **5**, 15336; doi: 10.1038/srep15336 (2015).

## Supplementary Material

Supplementary Information
