# Supplementary material for: The cell competition-based high-throughput screening identifies small compounds that promote the elimination of RasV12-transformed cells from epithelia
Source: Sci Rep. 2015 Oct 20;5:15336. doi: 10.1038/srep15336 (PMC4612300; doi:10.1038/srep15336)
Supplement: Supplementary Information [file srep15336-s1.pdf]

Supplementary Information

The cell competition-based high-throughput screening identifies small compounds that promote the elimination of RasV12-transformed cells from epithelia

Hajime Yamauchi, Takanori Matsumaru, Tomoko Morita, Susumu Ishikawa, Katsumi Maenaka, Ichigaku Takigawa, Kentaro Semba, Shunsuke Kon & Yasuyuki Fujita

Supplementary Figure S1
Supplementary Table 1
Supplementary Table 2

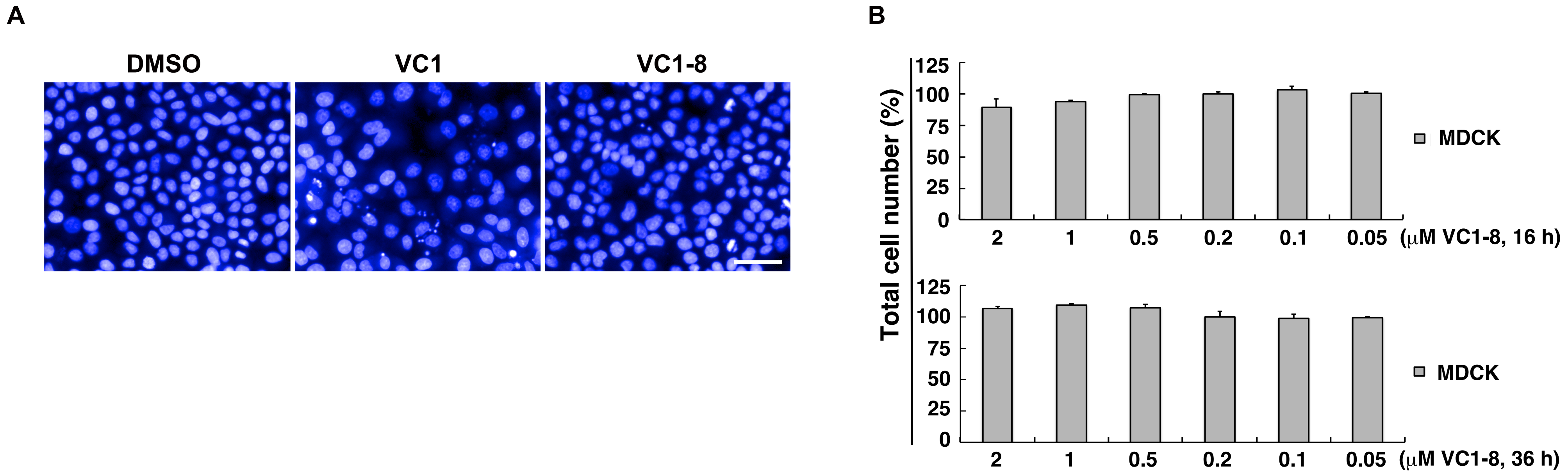

Supplementary Figure 1 l Cytotoxic effect of VC1 or VC1-8 on normal MDCK cells.
(A) Immunofluorescence images of the nucleus of MDCK cells that are treated with DMSO (left), 2 μM VC1 (middle), or 2 μM VC1-8 (right). Cells are stained with Hoechst 33342 (blue) after 36 h treatment. Scale bar: 50 μm.
(B) Dose-dependent effect of VC1-8 on the survival ratio of normal MDCK cells after treatment with VC1-8 for 16 h (top) or 36 h (bottom). Data are mean ± SD from three independent experiments. Values are expressed as a ratio relative to DMSO treatment.

Supplementary Table 1. Small Molecule Screening Data

Category	Parameter	Description
Assay	Type of assay	Cell-based co-culture assay
	Target	Promote the elimination of RasV12-transformed cells from the epithelium
	Primary measurement	GFP intensity of tetracyclilne-inducible MDCK-pTR GFP-RasV12 cells
	Key reagents	Tetracycline
	Assay protocol	Described in METHODS
	Additional comments	
Library	Library size	2,607 compounds
	Library composition	Known pharmacological activity
	Source	Several sources, including LOPAC^{1280} (Sigma-Aldrich) and Prestwick chemical compounds (Prestwick Chemical)
	Additional comments	Compounds were provided by Open Innovation Center for Drug Discovery, Tokyo University
Screen	Format	96-well, Optically Clear Bottom plates (CellCarrier, PerkinElmer)
	Concentration(s) tested	2 μM in 0.5% DMSO
	Plate controls	Positive control: DMSO and 10 μg/ml tetracycline<br>Negative control: DMSO alone
	Reagent/ compound dispensing system	Discovery Support Automatic Screening device Hornet-HTS (WAKO)
	Detection instrument and software	Operetta High Content Imaging System (PerkinElmer)
	Assay validation/QC	Z'-score of each assay was 0.548 ~ 0.930
	Correction factors	N/A
	Normalization	Normalization of GFP intensity per well was performed for compound treatment on each plate using the average of positive control (as 100% effect).
	Additional comments	Screened at Center for Research and Education on Drug Discovery, Faculty of Pharmaceutical Sciences, Hokkaido University
Post-HTS analysis	Hit criteria	GFP intensity outside the average ± 3SD of the positive control
	Hit rate	4.3 %
	Additional assay(s)	Secondary and tertiary screening assays (Described in METHODS)
	Confirmation of hit purity and structure	Hit compounds were repurchased and retested in triplicate
	Additional comments	

A

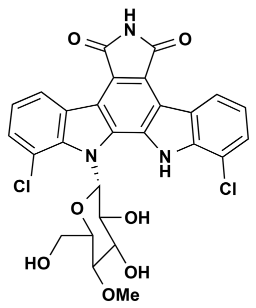

Rebeccamycin (=VC1)

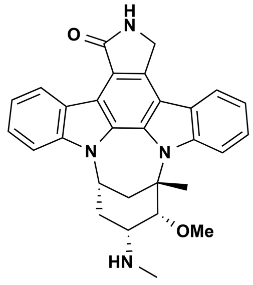

VC1-1

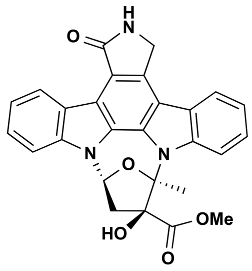

VC1-2

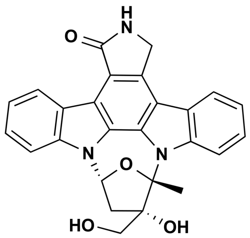

VC1-3

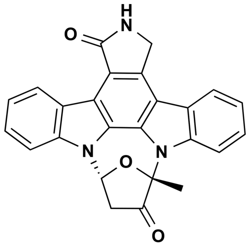

VC1-4

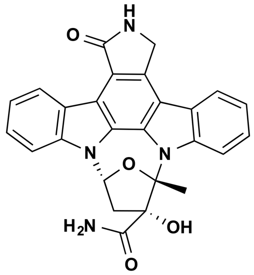

VC1-5

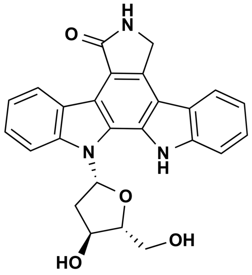

VC1-6

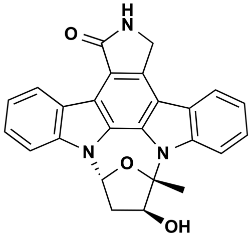

VC1-7

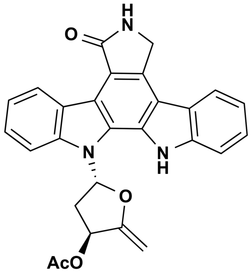

VC1-8

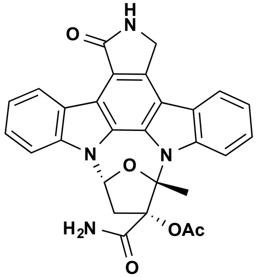

VC1-9

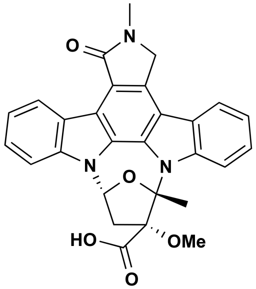

VC1-10

B

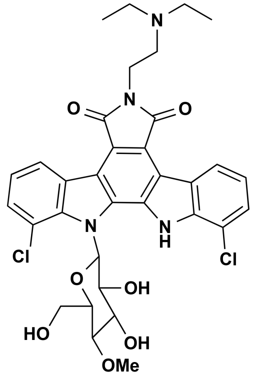

NSC-655649

Supplementary Table 2 | Structural formulae of Rebeccamycin and its analogous compounds.
(A) Structural formulae of analogue compounds of Rebeccamycin we have analyzed in this study.
(B) A structural formula of NSC65549.
